# Reprioritizing life: a conceptual model of how women with type 1 diabetes deal with main concerns in early motherhood

**DOI:** 10.1080/17482631.2017.1394147

**Published:** 2017-11-01

**Authors:** Ing-Marie Carlsson, Marie Berg, Annsofie Adolfsson, Carina Sparud-Lundin

**Affiliations:** ^a^ School of Health and Welfare, Department of Health and Nursing, Halmstad University, Halmstad, Sweden; ^b^ Centre for Person-Centered Care (GPCC), Institute of Health and Care Sciences, Sahlgrenska Academy, University of Gothenburg, Gothenburg, Sweden; ^c^ Department of Obstetrics and Gynecology, Sahlgrenska University Hospital, Gothenburg, Sweden; ^d^ School of Health Sciences, Örebro University, Örebro, Sweden

**Keywords:** Grounded theory, motherhood, type 1 diabetes mellitus, women’s health

## Abstract

**Purpose:** Becoming a mother is related to increased demands for women with type 1 diabetes mellitus, and more research is needed to identify their needs for support in everyday living. Thus, the aim of this study was to explore the main concerns in daily life in early motherhood for women with type 1 diabetes and how they deal with these concerns. **Method:** A grounded theory study was conducted in which 14 women with type 1 diabetes were interviewed individually 7 to 17 months after childbirth. **Results:** A conceptual model was identified with the core category “reprioritizing life”, and three related categories: adjusting to motherhood, taking command of the diabetes, and seeking like-minded women. Becoming a mother was a turning point towards a greater awareness and acceptance of prioritizing diabetes management and health, and thus, life. There was a gap in provision of diabetes care after birth and during the time of early motherhood compared with during pregnancy. **Conclusions:** Healthcare contacts already planned before delivery can promote person-centred care during the whole period from pregnancy to motherhood. Moreover, providing alternative sources for health information and peer support could improve the life situation during early motherhood.

## Introduction

Becoming a mother is one of the most central transitions in life (Meleis, Sawyer, Im, & Hillfinger, 2000), which may have multidimensional health implications and entails an opportunity to re-evaluate one’s life, including life goals, priorities, and preferences (Kaiser, Kaiser, & Barry, 2009). Women with type 1 diabetes mellitus (T1DM) encounter major challenges during their transitional period to motherhood (Rasmussen et al., 2013). This is related to the increased risks for the expected child, including malformations, accelerated gestational weight, and adverse outcomes during childbirth and the neonatal period (Inkster et al., 2006; Kinsley, 2007; Persson, Norman, & Hanson, 2009). To minimize the risks, the women during pregnancy have to struggle for normoglycaemia more or less 24 hours a day, 7 days a week, and are often filled with worries, pressure, and guilt for not living optimally (Berg & Honkasalo, 2000; Rasmussen et al., 2013). During pregnancy, they are usually provided frequent follow-up at the antenatal clinic, and their glycaemic control is thoroughly monitored in order to deliver a healthy baby.

When the child is born, a woman with T1DM encounters new challenges for both herself and her baby. Newborns to mothers with T1DM are more likely to develop neonatal hypoglycaemia and other morbidities, which often leads to treatment at neonatal care units (Ringholm, Mathiesen, Kelstrup, & Damm, 2012; Sparud-Lundin, Wennergren, Elfvin, & Berg, 2011). Breastfeeding is strongly encouraged due to its benefits for the mother and her baby (Ringholm et al., 2012; Sorkio et al., 2010). However, studies concerning breastfeeding rates and duration have shown contradictory results, with some indicating less breastfeeding in women with type 1 diabetes (Hummel et al., 2007; Schoen, Sichert-Hellert, Hummel, Ziegler, & Kersting, 2008), and others finding that lower rates were explained by maternal factors other than diabetes (Sorkio et al., 2010; Sparud-Lundin et al., 2011). In a previous case control study conducted by our research group, comprising mothers with T1DM and a reference group without diabetes, we found that both groups had high levels of confidence and assessed breastfeeding as a positive and important experience. However, to breastfeed was a greater challenge and struggle for the mothers with T1DM, due to unstable glycaemia that influenced daily life to a high degree. Although the mothers with T1DM reported high levels of confidence, they expressed more worry for own health, were more affected by disruptions in daily life, expressed a greater need to organize their life at both 2 and 6 months after childbirth, reported lower levels of general well-being, and were more tired than the reference group. A majority reported considerably more unstable glycaemia, especially during the first two months, and more hypoglycaemic episodes during the breastfeeding episode (Berg & Sparud-Lundin, 2012; Berg, Erlandsson, & Sparud-Lundin, 2012). A qualitative study has revealed that women with T1DM experience a disruption in care delivery after the child is born, although they face a challenging everyday life as new mothers with the additional burden of having diabetes (Sparud-Lundin & Berg, 2011). To develop sufficient health care for these women, there is a need to gain a deeper understanding of how they handle this vulnerable phase with less professional support than needed.

## Aim

The aim was to explore the main concerns in daily life in early motherhood for women with type 1 diabetes and how they deal with these concerns.

## Method

### Design

Grounded theory methodology aims to explain a general pattern of behaviour (Glaser & Strauss, 1967). In the present study a constructivist, grounded theory was chosen according to Charmaz (2014), who stresses that the “researcher is a part of what they study, not separate from it” (Charmaz, 2014, p. 320).

### Setting and participants

The women were recruited retrospectively from participants in a randomized controlled trial (RCT) evaluating web-based support carried out in Sweden for women with T1DM during pregnancy and up to 6 months after childbirth (MODIAB-Web). Women from both the intervention and control groups at three of the six included hospitals were asked to participate. The health care in the respective hospitals differed somewhat. In pregnancy all women with T1DM had frequent follow-ups at a hospital-based special antenatal care unit, which ended with a final follow-up around 6 weeks after birth. Parental leave in Sweden is financed by the Swedish Social Insurance Agency and the employer, and parents can use 480 days of paid parental leave for each child. Most of these days can be divided between the parents. The majority of mothers take full-time leave during the first months after birth (the Swedish Social Insurance Agency, 2010).

Based on a purposeful sampling procedure, eligible women were contacted by phone after data collection for the MODIAB-Web study was completed, that is, at least 6 months after childbirth, and asked about their willingness to participate in the present study. Of 18 women contacted, 4 declined and 14 agreed to participate. Interviews were conducted between 2014 and 2016. The participants had a median diabetes duration of 24 years (range 5–30). Their median age was 33 years (range 25–40); eight of them were primiparous and six were multiparous. One woman was single, and the others were living with the father of the child. Most women had a university degree (n = 8), five had completed secondary school, and one primary school.

### Data collection and analysis

The interviews were conducted 7–17 months after childbirth by three different researchers at a place chosen by the women. Five interviews were conducted by telephone due to distance. Each interview started with open-ended questions such as “Could you please tell me how you would describe your situation as a new mother with type 1 diabetes and how you handle your situation as a new mother with type 1 diabetes?” In accordance with the methodology, follow-up questions were posed, based on emergent data from previously analysed interviews (Corbin & Strauss, 2008). The interviews lasted 25–60 min and were audiotaped and transcribed verbatim.

The analysis process was started during data collection by summarizing each interview in a narrative memo to capture contextual content and to direct focus for the following interviews. The researchers discussed the preliminary findings after conducting five interviews, and then again after conducting a total of 10 interviews. In the following four interviews, specific focus was directed to emergent findings such as the meaning of breastfeeding, available support, and penetration of the strategies to manage the main concerns that had emerged during the first 10 interviews. During the further analysis process a constant comparison method was carried out according to Charmaz (2014), including initial coding in which the transcribed interviews were read line by line to capture incidents with relevance for the aim. Generated incidents were coded as condensed meaning markers of participants’ speech. These markers were written as gerunds and were held close to the data. During reading, questions were asked concerning the data to keep focus and guidance in the analysis: “*What is this study about?”* “*W* *hat is the main concern?”* and “*H* *ow is the main concern resolved?*” After initial coding, the focused coding followed with synthesizing codes. The coded incidents were compared, and patterns were discovered and pieced together according to similarities into conceptual explanations that fit and had relevancy to the aim of the study. Once the core category had emerged, the final coding step, that is, theoretical coding, started, which means saturation of properties of the constructed set of concepts. During the whole analysis process, a constant comparison of data was used to refine the data, and relations, interrelations, and concepts were further documented in memos. The final step was sorting of memos that were used and integrated into a conceptual model.

### Ethical considerations

The study was approved by the Regional Ethics Board (Dnr 775–13). Prior to the data collection, all of the mothers were given both written and oral information about the study. They were told that participation was voluntary and that they could withdraw their participation without further explanation at any time. Moreover, they were also guaranteed confidentiality, in accordance with good practice and research ethics in adherence to the principles of the Declaration of Helsinki (World Medical Association, 2013). The participants gave written consent to participate, and chose the timing and place of the interviews themselves.

## Findings

### The core category: reprioritizing life

The analysis found a core category, reprioritizing life, emerging as the main concern in early motherhood for the women with T1DM. Reprioritizing meant that becoming a mother and having chronic diabetes raised an awareness that life was fragile. This awareness often started during pregnancy and increased further after birth. Before pregnancy, the women could live and manage their diabetes as they wanted, since the diabetes affected only themselves. Then, they realized that they had another life to care for. During pregnancy the foetus was in the uterus and became affected by all abnormal glycaemia, and after birth the child needed a healthy mother. This strengthened awareness became a turning point in life, and served as an existential driving force, to manage the diabetes and to start taking better care of themselves, to stay healthy, and to avoid adverse effects and premature death.

I didn’t take care of it [diabetes] at all. But once I had children, it became very important, because it wasn’t just about me, anymore. It becomes very much that way. I’ve completely changed. Now, I really have to take care of myself. Because I want to be there for him for a long time, and take as good care of him as possible, by myself. (Interview person (IP) 1)

Reprioritizing life also concerned managing everyday life with its numerous hypoglycaemic episodes, which was, at least in the first period after birth, a big project entailing a constant struggle to reach glycaemic stability. To manage the diabetes was time consuming. The glycaemic levels fluctuated as a response to reduced needs of insulin, to hormonal changes, and to breastfeeding. Consequently, everyday life required a balancing act of prioritizing resources, working on what mattered most, and organizing daily life between one’s own needs of self-care due to diabetes management versus the desire of caring for the child’s needs.

It’s annoying that I now have to take my insulin, too… “You need to sit here and scream because Mommy has to give herself some insulin.…” I don’t like that, and of course I have to say, “We have to eat now”, but of course, he has to eat first, so I have to wait to give myself my shot, even though my level is really high, because I want him to have his food first, so at least he’ll be happy … so my blood sugar will have to be a bit high for a bit longer or something. That can be a bit challenging, because you know it’s not good for your blood sugar to be high. It becomes a stress factor. (IP 6)

Thus, reprioritizing life involved a reflection with reformulated incentives: the mothers had to sort themselves out first, before the child, when prioritizing day to day. Otherwise, there was a risk of hypoglycaemia that could affect their mood negatively, and thereby influence their child’s well-being. In a worst-case scenario, glycaemic instability could cause unconsciousness, risking both their own and the child’s health. This act of prioritizing and reformulating incentives was related to feelings of constant demands and self-blame, and took attention and concentration that was both time and energy consuming, which drained the women.

**Figure 1.**
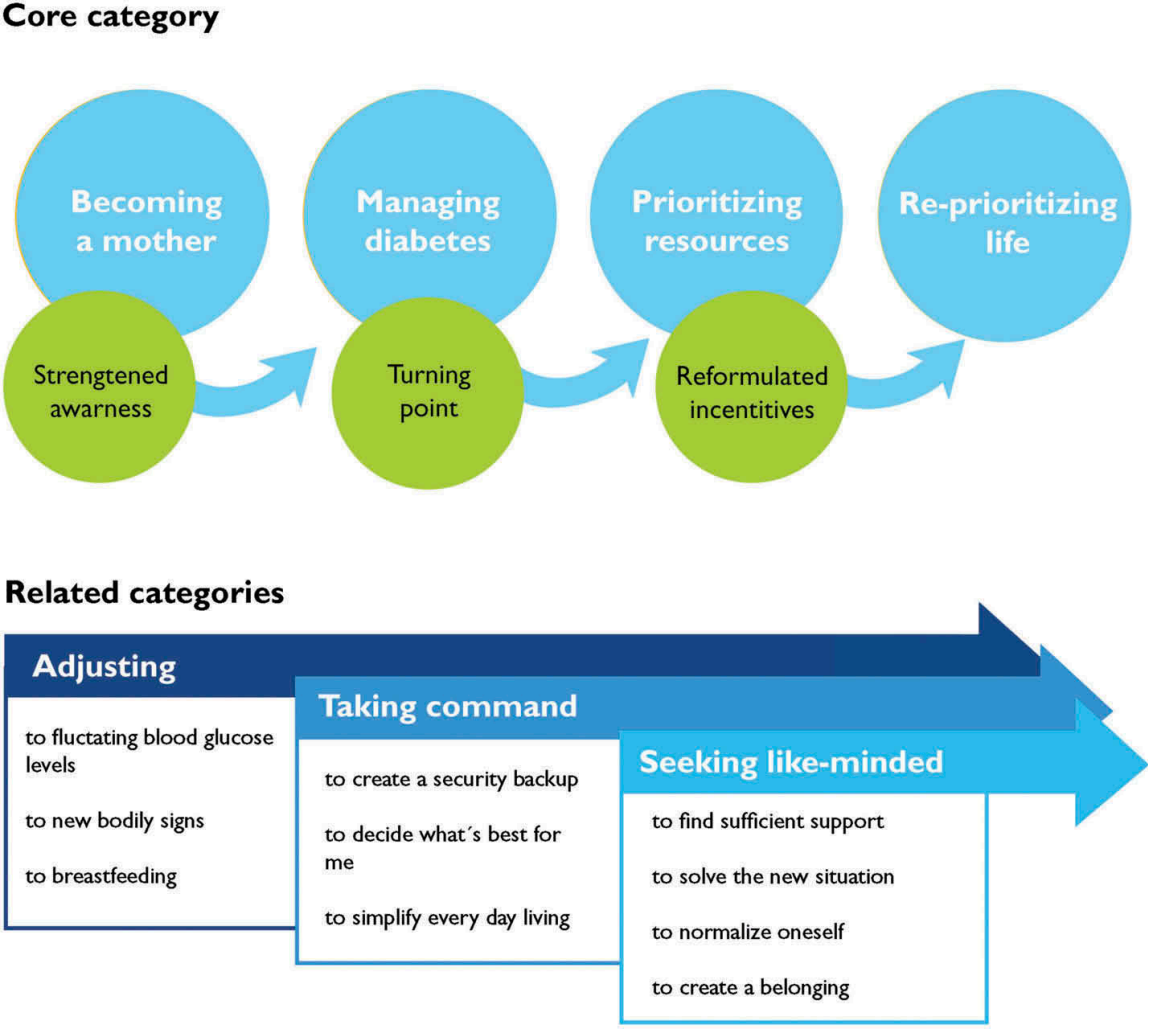
Conceptual model of the main concerns in early motherhood for women with type 1 diabetes and how they act to resolve their main concerns.

Three core-related categories conceptualize strategies that emerged and proceeded in a parallel process: adjusting to motherhood, taking command of the diabetes, and seeking like-minded women. Altogether, the core category and the three related categories form a conceptual model of the main concerns in early motherhood for women with type 1 diabetes and how they acted to resolve their main concerns (Figure 1).

## Core-related strategies for handling the main concern, “reprioritizing life”

### Adjusting to motherhood

The process of reprioritizing life started with adjusting to motherhood, a strategy that comprised handling and problem-solving both in the new life as a mother and in adjusting the diabetes management to this situation. It was a complex period involving tuning in to physiological and psychological bodily changes, establishing breastfeeding, and handling fluctuating blood glucose levels. Tuning in to the changed body consisted of being sensitive to new bodily signs as well as getting to know the body’s capacity after birth, simultaneously with managing the diabetes.

After birth the women experienced a radical reduction of insulin need. Especially in the very first days after birth it was common that the blood glucose levels fluctuated rapidly and often constituted hypoglycaemic episodes. Insulin doses had to be dramatically reduced, often to less than pre-pregnancy levels.

I had quite a lot of insulin in late pregnancy, but then, yes, just basically the minute he was born, I had to cut down a lot on insulin doses. (IP 13)

The process of bodily change was confusing and led to the diabetes being experienced as unfamiliar. Early after birth, a number of maternal as well as neonatal obstacles were faced in relation to breastfeeding. In some cases the problems started right after birth and were related to the fact that most babies needed extra feeding, sometimes intravenous glucose, related to hypoglycaemic episodes. This, in turn, affected the child’s ability to suck. Some mothers had needs for extra monitoring after a more complicated childbirth, such as an urgent Caesarean section or preeclampsia. This often led to separation of mother and child postpartum, which could further hinder establishment of breastfeeding.

Running back and forth when you’ve just had an episiotomy and everything … because it would have been much simpler if someone had been able to say, “It looks like he’s waking up. Do you want to get up and see if you can nurse him?” instead of you getting up and someone’s sitting there saying, “Well, he was hungry and now we’re feeding”. (IP 10)

The blood glucose fluctuations demanded the women’s attention and also created fear and feelings of uncertainty related to the responsibility for the baby’s safety, and also to their own health. The need to continuously adjust to fluctuating blood glucose levels created heightened preparedness, always being a step ahead in buffering oneself with carbohydrates such as bananas and sweet drinks. Often there was a need to keep a higher blood glucose level, which in turn could render feelings of guilt, and self-criticism for not managing the diabetes in an optimal way.

I thought it was awful at first, but I was never alone then. I think I would have been really scared if I had been alone…. Plus, I know if it hadn’t gone away I would probably have stopped nursing. Because my blood sugar could go from, like, 7 to 1.5 in a minute, when I started to nurse. My body couldn’t keep up, and didn’t react until I was at less than 2. (IP 5)

The need to adjust to the early phase of motherhood furthermore meant interrupted sleep causing tiredness, with adoption of new routines related to breastfeeding and the child’s other needs. The required attention to the diabetes was in this situation perceived as an additional burden taking time and focus from the child.

It’s of course something new that puts you even lower, plus lack of sleep on top of that and everything else. But diabetes lies there sucking energy out of you every day of your life, and of course when you are already fragile it sucks even more. (IP 3)

The challenges related to breastfeeding and fluctuating blood glucose levels drained the women’s energy and sometimes led to questioning whether it was worth it. Most of them wanted to breastfeed as was considered the best for the baby, and really tried hard to make it work. When it worked out well it somehow normalized life, which otherwise was overshadowed by the diabetes. Breastfeeding also competed with the women’s needed routines such as for regular meals, and, when these needs could not be fulfilled, it affected their blood glucose stability and created more urgent episodes of tackling abnormal blood glucose levels. One woman compared the breastfeeding effect on blood glucose with taking a walking tour in the mountains. Sometimes it was difficult for the women to prioritize their own health and well-being in relation to the demands of breastfeeding, and, consequently, it could result in termination of breastfeeding. Not being able to breastfeed was associated with feelings of failure and sadness, and termination was considered as a defeat, since they really tried to make it work.

The women expressed a need to be more prepared for this situation after childbirth and expressed a lack of knowledge on how breastfeeding affected diabetes and diabetes management, and this was especially important when they had their first child.

The first time I nursed, my blood sugar dropped really low. I didn’t know about that—no one had told me that it takes so much energy that you get low blood sugar. I was, like, living on bananas to get my blood sugar up, all the time. Finally, I couldn’t take it anymore. I had this huge headache all day and all night, from these blood sugar drops all the time, so I stopped nursing. (IP 2)

### Taking command of diabetes

To be able to resolve their main concern, reprioritizing life, the women needed to take command of their diabetes and achieve glycaemia goals. Taking command was a strategy that was partly self-chosen, knowing that nobody else could handle the situation and that the responsibility was theirs. They were the ones who were responsible for taking charge of and controlling the diabetes.

Don’t let it be this huge monkey on your back. Take control, get your diabetes on a leash. (IP. 7)

The strategy for taking command consisted of deciding what was best for themselves, creating a security backup, and getting technical tools that simplified everyday living. For some women, becoming a mother was a wake-up call: from now on they had to accept their diabetes and run a stricter regime, taking responsibility for themselves and their self-care.

It’s a matter of taking the time you need, taking time for yourself, taking time to eat and make sure … that you don’t think only about your child: you really do have to take care of yourself! (IP 8)

Creating a security back-up was accomplished by arranging safeguarding assistance from others who could take over, if needed. This gave a sense of assurance and was important for both themselves and their child, especially during the first weeks after birth. Having someone nearby if they failed to balance the demands of diabetes and motherhood was essential to be able to prioritize themselves. It consisted of having someone to call when an urgent need occurred, to get assistance with serving food if their blood glucose should drop quickly, and also gave them an extra hand with the child when energy was lacking.

Because I was always afraid when I was alone and nursing her, that I would feel it coming on. But when my partner was home I wasn’t as afraid, because he would always sit with us when I nursed, so he was ready and able to … and he helped me get some sugar when I sat with her. But when I was alone, I would automatically forget, and someone else would have to take her … and try to calm her down. So I was sometimes a bit scared my blood sugar would be too low just when I was about to start nursing. (IP 11)

Taking command was also expressed as a necessary strategy that was forced upon them due to lack of professional support after birth. During pregnancy and childbirth the women had access to frequent contacts with professionals who had expertise in diabetes management. After discharge from the maternal ward the women had mostly one follow-up for antenatal care, and it took a while before they had their first visit for their regular diabetes care. Instead, their meeting with healthcare professionals was related to the child health check-up system, which did not formally include checking the mothers, that is, the women themselves.

I haven’t told her [the nurse at the child care centre] that I … fell asleep, or that in the beginning I fell asleep a few times while nursing. Because I don’t know how knowledgeable she is in handling such issues…. It sort of seems like if you wanted to solve it, you would have to go into the details, check my blood sugar…. You go for check-ups there more frequently than to the diabetes clinic. (IP 4)

The women were familiar with dealing with their diabetes, but not in this new situation with both physical and mental changes due to motherhood. They lacked support from their ordinary diabetes care team; there was a gap in health care after childbirth. The lack of healthcare support gave them a feeling of being abandoned and left in “no-man’s land”, when no one in the health sector took responsibility for those with diabetes in early motherhood. Thus, it was necessary that they take command, to make decisions about what was best for them and thereby manage their situation. It would have been preferable if there had been established check-ups with the diabetes professionals starting directly after birth.

The strategy of taking command to simplify and make everyday life easier included using technical tools such as continuous glucose monitoring (CGM), and having an insulin pump, which for some women was integrated with CGM. Women who had given birth before compared their experiences of having access to more modern technical devices during this childbearing period. Such tools gave greater freedom and were considered to be of utmost importance, since the diabetes management was less time-consuming and gave time for other duties in daily life, especially taking care of the child.

Sure, I felt it more frequently in the first six weeks—definitely. But I was able to stop them in time, because I got warned by the pump. So … give everyone a pump. I’ve been extremely anti-pump over the years, because I don’t want to have something hanging off me. But … I have completely changed on that count! It’s like it made … everything has become much more natural. Just … yeah, the pregnancy was so much easier (than my previous ones), with this—and then to be able to go in and check, and just note, like, “Right, oh dear, between two and six”. I’m always a bit too high. But then I need to raise it. (IP 10)

### Seeking like-minded women

The third strategy related to “reprioritizing life” was to seek like-minded women, thus, other mothers in the same situation. To live with type 1 diabetes permeated everyday life, and even more in early motherhood. The women expressed the feeling that they were diabetics who had become mothers, rather than mothers with diabetes. This mirrored a self-image of being different and with different needs from mothers without diabetes. The information they had got from healthcare professionals about the episode as a new mother with diabetes was considered as insufficient for them. They lacked knowledge for being in problematic situations. Consequently, they developed their own expertise by trial and error, and they continually learned by adjusting and adapting to the new circumstances.

A strategy used was to seek like-minded peers, which involved searching for sufficient and relevant information that was more directed towards their particular problems related to the diabetes; for example, how to handle fluctuating blood glucose levels and insulin doses, or how to handle fatigue. The women found like-minded peers through different sources such as pregnancy groups or on different websites. Finding and communicating with a like-minded person in the same situation as them was crucial and helped them during early motherhood to problem solve and prioritize the dual role. Thereby, finding like-minded women was a strategy that facilitated and improved the women’s ability to perform self-care.

Finding a like-minded woman was of importance, because it confirmed that they were not alone; instead, they were one of many others who shared the same experiences. Recognizing themselves in each other’s stories created a sense of belonging and confirmed that what they had experienced was normal for those like themselves who had diabetes and had become mothers.

This diabetes buddy I’ve met, who is a new mother herself, it’s marvellous! I was on cloud nine when I got back from our walk. She got in touch with me because she was pregnant and needed support, but it was almost like after our first walk I thought I’ve totally talked her ear off, because I had this huge pent-up need. (IP 7)

The women expressed a constant feeling of being different from other mothers, that no one understood their special situation, and this rendered a feeling of being the loneliest in the world. Finding like-minded peers was a strategy that solved this problem. To improve daily life as a new mother with diabetes, it was suggested that an e-mail list signed by those pregnant women who agreed to it during pregnancy could facilitate establishing contact with somebody else in the same situation. It could also be possible to arrange a closed group on the internet by themselves, which was preferred to an open forum.

I had to go for walks with healthy mums [in a mums’ group], which was actually quite odd. It was nice to get to know other mums, but our situations, our experience, were actually so different and…. Most of them were nursing their babies, and I sat there bottle-feeding mine, so I felt pretty alone. I could really use a support group for diabetes. Yeah, a platform, I’m thinking. Of course there’s Facebook—that kind of diabetes group—but I don’t feel at home with them. Yeah, I’ve always looked for something I could make work but never found it, so that’s something I’ve missed. (IP 12)

## Discussion

This interview study show that women with type 1 diabetes had to reprioritize their everyday lives in early motherhood; their incentives were reformulated due to new prioritizations related to their own and the child’s needs. The findings on how they solve their main concerns in daily life during this vulnerable phase contribute new knowledge to this sparsely researched field. Healthcare professionals in both antenatal and diabetes care can be expected to benefit from the deeper understanding acquired in this study.

Adjusting to motherhood entailed a growing awareness that the women’s former experiences of having diabetes were no longer fully valid. After having struggled with the strict blood glucose levels during pregnancy, for the sake of the child, they now became aware that they need to stay healthy in order to be there for their child, from both a short- and a long-term perspective. During this phase, taking care of the newborn while simultaneously struggling with unstable glycaemia during the first months must be considered as extraordinary demands on a new mother, especially when they want to and do breastfeed. An earlier study (Stage, Norgard, Damm, & Mathiesen, 2006) found differences in hypoglycaemia rates in exclusively and not exclusively breastfeeding mothers four months after childbirth. A more recent study (Achong, McIntyre, Callaway, & Duncan, 2016) also found that breastfeeding women with T1DM had similar hypoglycaemia but lower glucose variability than women who did not breastfeed, as measured with CGM 2–4 months after childbirth. Suckling was shown to reduce maternal glucose levels but did not cause hypoglycaemia in most episodes (85%). This is not in line with other studies with self-reports of more frequent hypoglycaemic episodes and fluctuating blood glucose levels (Berg & Sparud-Lundin, 2012; Edwards, Speight, Bridgman, & Skinner, 2016; Sparud-Lundin & Berg, 2011). This can be explained by the fact that Achong et al. (2016) and Stage et al. (2006) did not measure blood glucose levels in the early postpartum period, for example, before 2 months after childbirth. Although not measured by CGM, other studies have shown unstable glycaemic control and decreased insulin requirements due to increased maternal glucose consumption during the first weeks postpartum (Riviello, Mello, & Jovanovic, 2009).

In line with what was described in our study regarding the benefits of having technical tools that support daily life, it seems relevant to advocate for the availability of getting CGM advice in the early postpartum period. This would be to assist the mother’s adjustment to rapid changes in blood glucose levels, and especially to avoid hypoglycaemic episodes, as they interfere with taking care of the baby, including the child’s security. The women’s heightened awareness of the need for control, together with a reassuring effect of early alarms in case of a trend towards hypoglycaemia, might support these women in taking command of their diabetes. Fallon and Dunne (2015) claim that fundamental needs for women with type 1 diabetes are those related to safety and security. Since professional support is not available 24 h a day, particularly during early motherhood, technical devices can assist in continuously monitoring the unfamiliar and unpredictable blood glucose levels. This could be time and energy saving and might decrease the burden of daily self-management, potentially leading to better well-being and less exhaustion for these women. This is in line with what was found in a previous study by our research group (Berg & Sparud-Lundin, 2012; Berg et al., 2012).

Beyond technical devices for diabetes management, healthcare providers and peers could play a crucial role in supporting these women during early motherhood. The form of antenatal care provider has been found to influence the intention to breastfeed in women with diabetes (Finkelstein et al., 2013). For women in our study the frequent contacts with the antenatal care providers during pregnancy were abruptly interrupted when the child was born. Other healthcare professionals, either during the early postpartum care or after discharge, did not replace their supportive role. During a period of experiencing a more unfamiliar diabetes than ever before, and with reformulated incentives for taking care of themselves simultaneously with caring for the baby, most of the women in our study felt left with the responsibility. As the healthcare system did not support them sufficiently during early motherhood, they searched for support from other women in similar conditions. This is in line with the findings of Edwards et al. (2016), who made an extensive qualitative exploration of 200 electronic interactions between women with type 1 diabetes, from planning pregnancy, throughout pregnancy, and into early motherhood. The communication was retrieved from a free Internet-based counselling service and psychosocial support provided by a non-profit organization mainly for people with diabetes. Diabetes-specific distress was most frequent during “contemplation”, for example, when considering pregnancy, followed closely by the phase of motherhood. Depressed mood peaked during motherhood, and partners were not always able to understand their experiences. The authors conclude that contacts with other women who have experiences of pregnancy and motherhood seem to offer assurance not provided by their healthcare providers (Edwards et al., 2016). Although healthcare providers can assist in advising on how to make adjustments in insulin doses and so forth in order to balance the juggling of blood glucose, peers with diabetes are more likely to help prioritize the dual role in early motherhood.

### Methodological limitations

Although the findings in this study resonate with other research, and by that demonstrate transferability, some limitations need to be acknowledged. First, the question of selection bias is always present in research studies. However, the effort to achieve a varied sample of women with type 1 diabetes with respect to experiences both of being a first-time mother and of having given birth earlier increases external validity. Second, socioeconomic differences regarding educational levels, social welfare systems (for paid parental leave) and healthcare systems, as well as varying sociocultural attitudes towards breastfeeding, can have an impact on the transferability of the findings to other societies.

## Conclusion and clinical implications

Having diabetes and being in the early period after giving birth to a child is demanding and challenging. The healthcare system needs to support these women, especially first-time mothers. After the extraordinary health care provided during pregnancy, it would be highly relevant and important to at least phase out the frequent support more gradually after the childbirth, not least because of the new challenges faced in relation to diabetes management in early motherhood. Therefore, we propose that a woman with type 1 diabetes should have planned and frequent contacts with her diabetes nurse directly after childbirth, preferably arranged beforehand. To secure the diabetes management support, telephone follow-up once or twice a week could be relevant during this extra-demanding period, as well as accessibility to technical support for facilitating daily self-management, if wanted. Moreover, providing these women with alternative sources for health information and peer support beyond the antenatal and diabetes care has the potential to improve their life situation during motherhood. This needs to be further investigated in upcoming studies.
